# Frailty, Comorbidity, and Associations With In-Hospital Mortality in Older COVID-19 Patients: Exploratory Study of Administrative Data

**DOI:** 10.2196/41520

**Published:** 2022-12-12

**Authors:** Johannes Heyl, Flavien Hardy, Katie Tucker, Adrian Hopper, Maria J M Marchã, Annakan V Navaratnam, Tim W R Briggs, Jeremy Yates, Jamie Day, Andrew Wheeler, Sue Eve-Jones, William K Gray

**Affiliations:** 1 Department of Physics and Astronomy University College London London United Kingdom; 2 Getting It Right First Time programme National Health Service England and National Health Service Improvement London United Kingdom; 3 Innovation and Intelligent Automation Unit Royal Free London National Health Service Foundation Trust London United Kingdom; 4 Guy’s and St Thomas’ National Health Service Foundation Trust London United Kingdom; 5 Science and Technology Facilities Council Distributed Research Utilising Advanced Computing High Performance Computing Facility University College London London United Kingdom; 6 University College London Hospitals National Health Service Foundation Trust London United Kingdom; 7 Royal National Orthopaedic Hospital National Health Service Trust London United Kingdom; 8 Department of Computer Science University College London London United Kingdom

**Keywords:** COVID-19, coronavirus, SARS-CoV-2, frailty, comorbidity, mortality, death, hospitalization, hospital admission, hospitalisation, patient, age, sex, ethnicity, disease, hospital, cancer, heart, heart failure, weight loss, weight, renal disease, support, geriatric, older adult, elder, descriptive statistics, machine learning, model

## Abstract

**Background:**

Older adults have worse outcomes following hospitalization with COVID-19, but within this group there is substantial variation. Although frailty and comorbidity are key determinants of mortality, it is less clear which specific manifestations of frailty and comorbidity are associated with the worst outcomes.

**Objective:**

We aimed to identify the key comorbidities and domains of frailty that were associated with in-hospital mortality in older patients with COVID-19 using models developed for machine learning algorithms.

**Methods:**

This was a retrospective study that used the Hospital Episode Statistics administrative data set from March 1, 2020, to February 28, 2021, for hospitalized patients in England aged 65 years or older. The data set was split into separate training (70%), test (15%), and validation (15%) data sets during model development. Global frailty was assessed using the Hospital Frailty Risk Score (HFRS) and specific domains of frailty were identified using the Global Frailty Scale (GFS). Comorbidity was assessed using the Charlson Comorbidity Index (CCI). Additional features employed in the random forest algorithms included age, sex, deprivation, ethnicity, discharge month and year, geographical region, hospital trust, disease severity, and International Statistical Classification of Disease, 10th Edition codes recorded during the admission. Features were selected, preprocessed, and input into a series of random forest classification algorithms developed to identify factors strongly associated with in-hospital mortality. Two models were developed; the first model included the demographic, hospital-related, and disease-related items described above, as well as individual GFS domains and CCI items. The second model was similar to the first but replaced the GFS domains and CCI items with the HFRS as a global measure of frailty. Model performance was assessed using the area under the receiver operating characteristic (AUROC) curve and measures of model accuracy.

**Results:**

In total, 215,831 patients were included. The model using the individual GFS domains and CCI items had an AUROC curve for in-hospital mortality of 90% and a predictive accuracy of 83%. The model using the HFRS had similar performance (AUROC curve 90%, predictive accuracy 82%). The most important frailty items in the GFS were dementia/delirium, falls/fractures, and pressure ulcers/weight loss. The most important comorbidity items in the CCI were cancer, heart failure, and renal disease.

**Conclusions:**

The physical manifestations of frailty and comorbidity, particularly a history of cognitive impairment and falls, may be useful in identification of patients who need additional support during hospitalization with COVID-19.

## Introduction

Various studies have been conducted to look at the factors that contribute the most to poorer outcomes for people with COVID-19. In both community-based and hospital-based studies, age has consistently been found to be the strongest predictor of mortality in people with COVID-19 [1]. However, distinguishing between the effects of chronological age and the effects of age-related changes in health status linked to frailty and comorbidities could improve patient-centered care and health care resource allocation [2-5].

Many previous studies of frailty in COVID-19 have used the Clinical Frailty Scale (CFS) to assess frailty status [6-11]. CFS-assessed frailty has been found to be consistently associated with mortality risk in COVID-19 patients [12]. However, as a clinical tool, the CFS score is usually not recorded in large databases, and these studies tend to be of relatively small cohorts. A recent systematic review of studies using the CFS identified a strong link between frailty and mortality but noted that most studies were at high risk of bias and suggested that further studies were warranted [13]. Larger studies have been conducted, but have often focused on specific cohorts of patients, such as those in critical care [14,15].

A number of tools have been developed to identify frailty and comorbidity from large administrative databases, including some developed using artificial intelligence algorithms [16,17]. A recent review [12] identified 5 such tools, including the electronic Frailty Index [18] (for use in primary care), the Hospital Frailty Risk Score (HFRS) [19], the Global Frailty Scale (GFS) [20], and the Charlson Comorbidity Index (CCI) [21]. Such tools rely on coded diagnostic data and may help provide insights beyond those that can be obtained from smaller clinical studies of COVID-19 patients.

The aim of this study was to assess the potential of an administrative database of patients aged 65 years or older to explore the relationship between frailty and comorbidities (defined using coded diagnostic data) and COVID-19 in-hospital mortality. We used machine learning algorithms to analyze the data. Machine learning offers a flexible approach to exploratory analysis, as it makes no a priori assumptions about the hierarchy of variables or their relationships. This allowed us to assess the relative importance of the various frailty and comorbidity features in relation to in-hospital mortality. It is particularly important to be able to identify the relative importance of these frailty and comorbidity features, which are typically long-term in nature, relative to admission-specific items.

## Methods

### Ethical Considerations

Ethical approval was not sought for the present study because it did not directly involve human participants. Consent from individuals involved in this study was not required for this analysis of the Hospital Episodes Statistics (HES) administrative data set. Guidance from National Health Service (NHS) Digital for the use and reporting of HES data for research purposes was followed, with anonymization to the level required by the ISB1523 Anonymisation Standard for Publishing Health and Social Care Data [22]. This study was completed in accordance with the Helsinki Declaration as revised in 2013.

### Study Design and Data Collection

This was a retrospective, exploratory analysis of HES data. HES data are collected by NHS Digital for all NHS-funded patients admitted to hospitals in England. Data are entered by trained clinical coders in each hospital trust; data collection and reporting are mandatory. The data collected include demographics, the nature and timing of admission and discharge, diagnoses, and procedures undertaken.

### Timing, Case Ascertainment, and Inclusion and Exclusion Criteria

We reviewed HES data for all completed episodes of hospital care in England with a discharge date from March 1, 2020, to February 28, 2021, that involved a diagnosis of COVID-19. We only considered completed episodes of care in which the patient had been discharged and their outcome (died or survived) was known. Patients aged <65 years were excluded. Cases of COVID-19 were identified using the International Statistical Classification of Disease, 10th Edition (ICD-10) codes (2019 version) U07.1 (ie, presence of COVID-19 has been confirmed by laboratory testing) and U07.2 (ie, clinical or epidemiological diagnosis of COVID-19 where laboratory confirmation is inconclusive or not available). The diagnoses were made either on admission or during the stay and could be primary or secondary. These 2 codes were created by the World Health Organization to code COVID-19 data [23].

Where a patient had multiple admissions during the study period, only the chronologically last admission was retained. This ensured that all admissions were independent of one another at a patient level and avoided biasing the data by including cases where the outcome was predefined by virtue of a subsequent admission.

### Outcomes

The outcome of interest was in-hospital mortality, as recorded by the Office for National Statistics. All data were available to us though NHS Digital and linked at a patient level using a pseudonymized patient identifier. An in-hospital death was recorded if the date of death was the same as or within 1 day of the hospital discharge date. Data on length of stay were also extracted and used to compare the relationship between these 2 patient outcomes.

### Features

#### Frailty/Comorbidity Features

The HFRS was categorized as none, mild, moderate, or severe for the descriptive analysis and as a continuous score in the machine learning algorithm [19]. The HFRS is calculated from 109 ICD-10–coded diagnoses during the index admission of any admission in the previous 2 years to give a weighted score. The HFRS gives a global assessment of frailty status and cannot be broken down into individual domains. It has been validated for use in a number of settings. [19,24-26]

The GFS defines 7 domains of frailty (dementia and delirium; mobility problems; falls and fractures; pressure ulcers and weight loss; incontinence; and anxiety and depression) based on ICD-10 codes for hospital admissions during the previous year [20]. The GFS is closely aligned with the key clinical subdomains of frailty and considers the impact of manifestations of frailty on functional ability. It was developed by considering the relationship between the frailty domains and long hospital stays, 30-day nonelective readmission, and in-hospital mortality. It has not been validated outside of the original development study. The domain of dependency/care was not used, as an exploratory analysis suggested that the 2 ICD-10 codes used to define it (Z74 and Z75) were used in HES to identify patients who had survived to discharge but could not be discharged due to an unmet social care need.

The CCI identifies 14 specific medical conditions identified as secondary diagnoses in the index admission and primary or secondary diagnoses in any admission during the previous year. The conditions are peripheral vascular disease, congestive heart failure, acute myocardial infarction, cerebrovascular disease, dementia, chronic pulmonary disease, connective tissue disease/rheumatic disease, peptic ulcer, liver disease (mild and moderate/severe), diabetes (with and without chronic complications), paraplegia/hemiplegia, renal disease, cancer (primary and metastatic), and HIV/AIDS [21]. It has been extensively validated [27].

An index admission diagnosis of obesity was based on ICD-10 code E66.

#### Non–Frailty/Comorbidity Features

Age was categorized as bands (65-69 years, 70-79 years, and 80 years or older) for descriptive analysis and as a continuous variable when input into the machine learning algorithm.

Sex was categorized as female or male.

Ethnicity was categorized as White, Black or Black British, South Asian or South Asian British, other Asian or other Asian British, mixed, or other. For a number of patients, an ethnicity category was not recorded. In these cases, the HES database was searched for the most recent prior hospital admission for the same patient where ethnicity had been recorded and this value was used.

The index of multiple deprivation (IMD) score (2019 version) was used to categorize relative deprivation. It is measured in England by assigning each of England’s 32,844 lower layer super output areas (LSOAs) a deprivation score calculated from a weighted average of 7 deprivation-related domains: income (22.5%), employment (22.5%), health deprivation and disability (13.5%), education or skills training (13.5%), crime (9.3%), barriers to housing and services (9.3%), and living environment (9.3%) [28-30]. The IMD score is reported as deciles in the descriptive analysis and used as a continuous variable in the machine learning algorithm.

Hospital trusts typically run between 1 and 4 NHS hospitals covering a geographically defined catchment.

NHS regions include London; the southeast, southwest, and east of England; the Midlands; the northeast and Yorkshire, and the northwest.

The individual ICD-10 codes recorded in the diagnostic record during the hospital stay were included as binary features.

### Data Analysis and Model Building

Data were analyzed using the Python programming language (version 3.9, Python Software Foundation). Descriptive statistics techniques were used to summarize the data in the covariate categories described above.

All machine learning models were developed using the scikit-learn library. Random forest classifiers were used to identify key covariates associated with in-hospital mortality. Random forest classifiers are ensemble classifiers that fit decision trees to portions of the data and average over all decision trees. This is of particular importance if a machine learning model is to provide useful information about the relationship between the features and the outcome variable to clinicians. Machine learning has an advantage over traditional statistical models because it does not make any assumptions about the nature of the model. Machine learning has shown benefits in analyzing health care data [31-33].

To identify the most important features for each model, we used the SHAP (Shapley additive explanation) feature importance method [34]. Feature importance values were calculated using TreeSHAP, an efficient estimation approach for tree-based models. The SHAP feature importance method allows for the identification of the nature of the relationship between the individual features and the output variable [35]. In a plot of SHAP values, each dot in the plot represents a patient. The dots are colored red or blue. The color of the dot represents the size of the feature relative to the range of values that feature can take, with red representing large feature values and blue low feature values. A positive SHAP value can be interpreted as meaning the feature is associated with in-hospital mortality. A negative SHAP value can be interpreted as meaning the feature is associated with the patient surviving to discharge. The features are ranked by the mean of the absolute value of the SHAP values.

Two different random forest models were constructed to classify patients according to mortality status, and their predictive accuracy was compared. The models differed in their choice of features. Model 1 included age, sex, deprivation, ethnicity, region, NHS trust, ICD-10 codes, the 14 CCI items, and the GFS domains. Model 2 included the same items as model 1, except the HFRS bands were added as a feature and the CCI items and the GFS domains were removed. The 2 models allowed a comparison of the performance of a model that included individual frailty domains and comorbidities (model 1) and one that included a single global measure of frailty. All listed variables were included in the final model, although only the most important features are described.

To avoid collinearity, features with a high degree of correlation (ie, a bivariate correlation coefficient >0.5) were excluded. The dementia item from the CCI and the dementia/delirium item from the GFS had a correlation coefficient of 0.6. As the GFS item had the broader definition, this was used as a covariate and the CCI item was excluded. No other items were excluded due to high correlation.

For data preprocessing, the data set was randomly split at a ratio of 70:15:15 into a training set, a testing set, and a validation set, respectively. All 3 data sets contained patients who had died and patients who had survived. The machine learning algorithm was trained on the training set and its performance was evaluated based on how well it could predict mortality in the test set. To ensure that the model did not simply classify according to the majority outcome (ie, survival), the training set was reduced further by randomly removing patients who had survived to ensure that there were an equal number of patients who had died and who had survived in the training set. This eliminated the effect of the class imbalance on the model performance and ensured that the model had sufficient exposure to patients who died. However, the test set on which the trained model was evaluated was not balanced, increasing the model’s external validity. The validation set was used to tune the hyperparameters of the random forest. There are several hyperparameters specific to the random forest classifier that can be tuned. The combination of hyperparameters with the highest area under the receiver operating characteristic (AUROC) curve was selected. The optimal hyperparameters were found by using the Bayesian optimization library. The hyperparameter ranges used are listed in Table S1 in Multimedia Appendix 1. These hyperparameters included the number of trees (n=112), the minimum samples per split (n=8) and the minimum samples per leaf (n=1). The AUROC curve was plotted as sensitivity versus 1–specificity [36].

Categorical variables were one-hot encoded. This involved creating a binary column for each value that the variable could take. For example, for NHS region, a patient treated in the Midlands would have a value of 1 in the Midlands column, but a value of 0 in the other regional categories. The algorithm for model 1 was used to construct a model of the relationship between length of stay and in-hospital mortality.

In the sensitivity analysis, the performance of the random forest classifier was compared to extreme gradient boosting (XGBoost) and multivariable logistic regression models.

Other than for ethnicity (see “Features”), missing data were relatively rare, and no attempt was made to impute missing values. Patients with missing data were omitted from the analysis. The number of missing values for each variable is given in Table 1.

**Table 1:** Demographic characteristics and in-hospital deaths of patients.

Characteristics			Number of patients (N=215,831)		In-hospital deaths (n=77,738)		Chi-square (*df)*			*P* value	
**Age band (years), n (%)**								4213.2 (2)			<.001
	65-69	27,401 (12.7)		6431 (23.5)							
	70-79	73,568 (34)		23,277 (31.6)							
	≥80	114,862 (53.2)		48,030 (41.8)							
**Sex^a^, n (%)**								1646.9 (1)			<.001
	Female	101,989 (47.3)		32,351 (31.7)							
	Male	113,826 (52.7)		45,382 (40)							
**Deprivation decile^b^, n (%)**								16.2 (9)			.06
	1 (most deprived)	25,053 (11.6)		8862 (35.4)							
	2	24,937 (11.3)		8679 (35.6)							
	3	23,320 (10.8)		8441 (36.2)							
	4	21,756 (10.1)		7884 (36.2)							
	5	21,044 (9.8)		7701 (36.6)							
	6	21,004 (9.7)		7732 (36.8)							
	7	20,149 (9.3)		7273 (36.1)							
	8	19,787 (9.2)		7212 (36.4)							
	9	18,764 (8.7)		6724 (35.8)							
	10 (least deprived)	17,012 (7.9)		6123 (36)							
**Region in England^c^, n (%)**								246.1 (6)			<.001
	East	22,934 (10.6)		9096 (39.7)							
	London	35,912 (16.6)		12,617 (35.1)							
	Midlands	44,590 (20.7)		16,072 (36)							
	Northeast and Yorkshire	34,850 (16.1)		12,187 (35)							
	Northwest	35,281 (16.3)		12,971 (36.8)							
	Southeast	29,562 (13.7)		10,554 (35.7)							
	Southwest	12,028 (5.6)		4085 (34)							
**Ethnicity^d^, n (%)**								46.1 (5)			<.001
	White	181,453 (84.1)		65,440 (36.1)							
	Black or Black British	5794 (2.7)		2108 (36.4)							
	South Asian or South Asian British	10,216 (4.7)		3910 (38.3)							
	Other Asian or other Asian British	2659 (1.2)		953 (35.8)							
	Mixed	963 (0.4)		342 (35.5)							
	Other	4484 (2.1)		1488 (33.2)							
**Disease severity, n (%)**											
	Pneumonia	144,206 (65.9)		66,323 (46.6)		18,757.8 (1)			<.001		
	Renal disease	55,155 (25.6)		29,353 (53.2)		9512.3 (1)			<.001		
	Blood clotting	6836 (3.2)		3017 (44.1)		201.8 (1)			<.001		
	Cardiology/circulation	4967 (2.3)		2529 (50.9)		489.7 (1)			<.001		
	Neurology	6986 (3.2)		3022 (43.3)		164.2 (1)			<.001		
	Digestive system	235 (0.1)		134 (57)		45 (1)			<.001		
	Sepsis	16,327 (7.5)		9534 (58.4)		3837.6 (1)			<.001		

^a^There were 16 missing values.

^b^There were 3545 missing values.

^c^There were 674 non–National Health Service providers.

^d^Not stated in 10,262 values.

## Results

The data extraction process resulted in a data set of 215,831 patients (Figure S1 in Multimedia Appendix 1). The crude mortality rate was 36% (77,738/215,831). The breakdown of patient numbers and the associated mortality rate is presented by age, sex, deprivation decile, region, ethnicity, and disease severity marker in Table 1 and by GFS domain and CCI item in Table 2. Higher in-hospital crude mortality rates were seen in older age groups, men, and in almost all comorbidity and frailty groups, except those with mild liver disease and anxiety or depression. There was no obvious relationship between in-hospital mortality and deprivation and a relatively modest difference between the different ethnic groups, with South Asian patients having the highest in-hospital mortality rate. The median length of hospital stay was 10 (IQR 5-20) days in patients who survived to discharge and 9 (IQR 4-17) days in those who died in hospital.

**Table 2:** Mortality rates by comorbidity/frailty measure. Significance was tested using the chi-square test with significance set at 5%. For each comorbidity and frailty item, the number of patients with the condition is given together with the number of in-hospital deaths.

Comorbidity/frailty items		Patients (N=215,831), n (%)	In-hospital deaths (n=77,738), n (%)	Chi-square (*df*)	*P* value
**Charlson Comorbidity Index**					
	Peripheral vascular disease	15,519 (7.2)	6663 (42.9)	358.9 (1)	<.001
	Congestive heart failure	42,370 (19.6)	20,433 (48.2)	3412.8 (1)	<.001
	Acute myocardial infarction	26,670 (12.4)	11,416 (42.8)	611.2 (1)	<.001
	Cerebrovascular disease	28,773 (13.3)	11,241 (39.1)	137.9 (1)	<.001
	Dementia	44,036 (20.4)	18,749 (42.6)	1098.1 (1)	<.001
	Chronic pulmonary disease	63,244 (29.3)	24,298 (38.4)	227.3 (1)	<.001
	Connective tissue/rheumatic disease	7867 (3.6)	2964 (37.7)	8.2 (1)	.004
	Peptic ulcer	1979 (0.9)	764 (38.6)	7.0 (1)	.008
	Mild liver disease	7402 (3.4)	2664 (36)	0 (1)	.92
	Moderate or severe liver disease	1706 (0.8)	975 (57.2)	344.9 (1)	<.001
	Diabetes without chronic complications	59,815 (27.7)	22,704 (38)	133.5 (1)	<.001
	Diabetes with chronic complications	7190 (3.3)	2864 (39.8)	43.2 (1)	<.001
	Paraplegia and hemiplegia	5667 (2.6)	2253 (40)	38.2 (1)	<.001
	Renal disease	55,652 (25.8)	24,947 (44.8)	2533.5 (1)	<.001
	Primary cancer	21,822 (10.1)	9764 (44.7)	864.1 (1)	<.001
	Metastatic carcinoma	8095 (3.8)	3675 (45.4)	378.3 (1)	<.001
	HIV/AIDS	72 (0.03)	19 (26.4)	7.4 (1)	.006
	Obesity	14,766 (6.8)	5222 (35.5)	5.4 (1)	.02
**Global Frailty Scale**					
	Dementia and delirium	76,669 (35.5)	32,011 (41.8)	1696.6 (1)	<.001
	Mobility problems	29,191 (13.5)	11,207 (38.4)	82.6 (1)	<.001
	Falls and fractures	81,805 (37.9)	31,957 (39.1)	530.7 (1)	<.001
	Pressure ulcers and weight loss	23,249 (10.8)	10,814 (46.5)	1245.5 (1)	<.001
	Incontinence	15,359 (7.1)	6095 (39.9)	96.4 (1)	<.001
	Anxiety and depression	25,268 (11.7)	8123 (32.1)	186.0 (1)	<.001

The training data set included 151,081 patients, the test data set included 32,374 patients, and the validation data set included 32,376 patients. Table 3 shows the performance of the random forest classifier on the test set in the 2 models developed. The best performing model was model 1, which included the GFS domains and CCI items and had an accuracy of 83%, an AUROC curve of 90%, and a true positive rate of 81%. Model 2 had slightly poorer performance, with an accuracy of 82%, an AUROC curve of 90%, and a true positive rate of 80%. The AUROC curve for model 1 is shown in Figure S2 in Multimedia Appendix 1.

**Figure 1:**
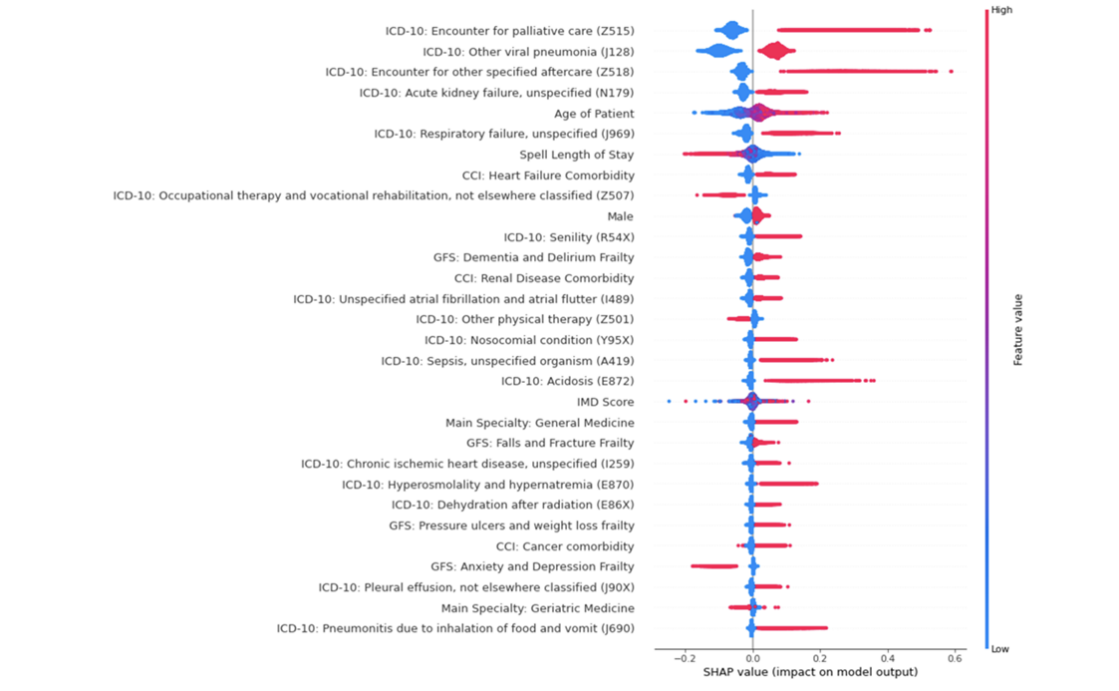
Shapley value dot summary plot for model 1. Each dot in the plot represents a patient. The x-axis indicates whether there is a positive or negative correlation between the value of the feature and its contribution to the model prediction of a patient dying. The color of the dot represents the size of the feature relative to the range of values that feature can take, with red representing large feature values and blue low feature values. The horizontal axis represents the association of the feature value with the outcome. A positive SHAP value means the feature is associated with mortality. A negative SHAP value means the feature contributes to the patient surviving to discharge. The features are ranked by the mean of the absolute value of the SHAP values. CCI: Charlson Comorbidity Index; GFS: Global Frailty Scale; ICD-10: International Statistical Classification of Disease, 10th Edition; IMD: index of multiple deprivation; SHAP: Shapley additive explanation.

**Figure 2:**
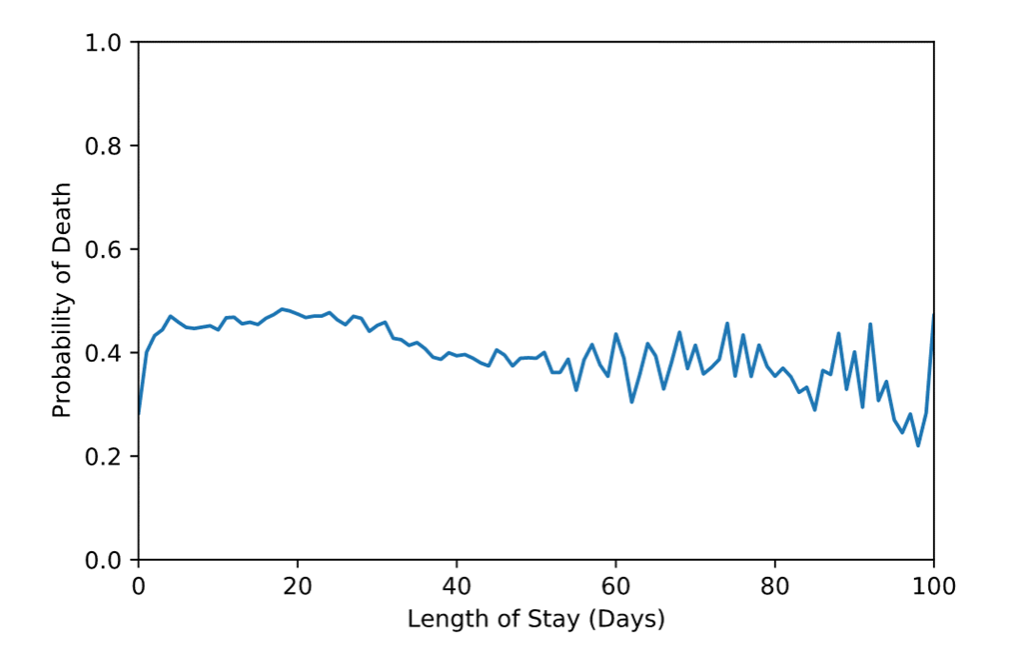
Plot of the predicted probability of death as a function of the length of stay.

**Figure 3:**
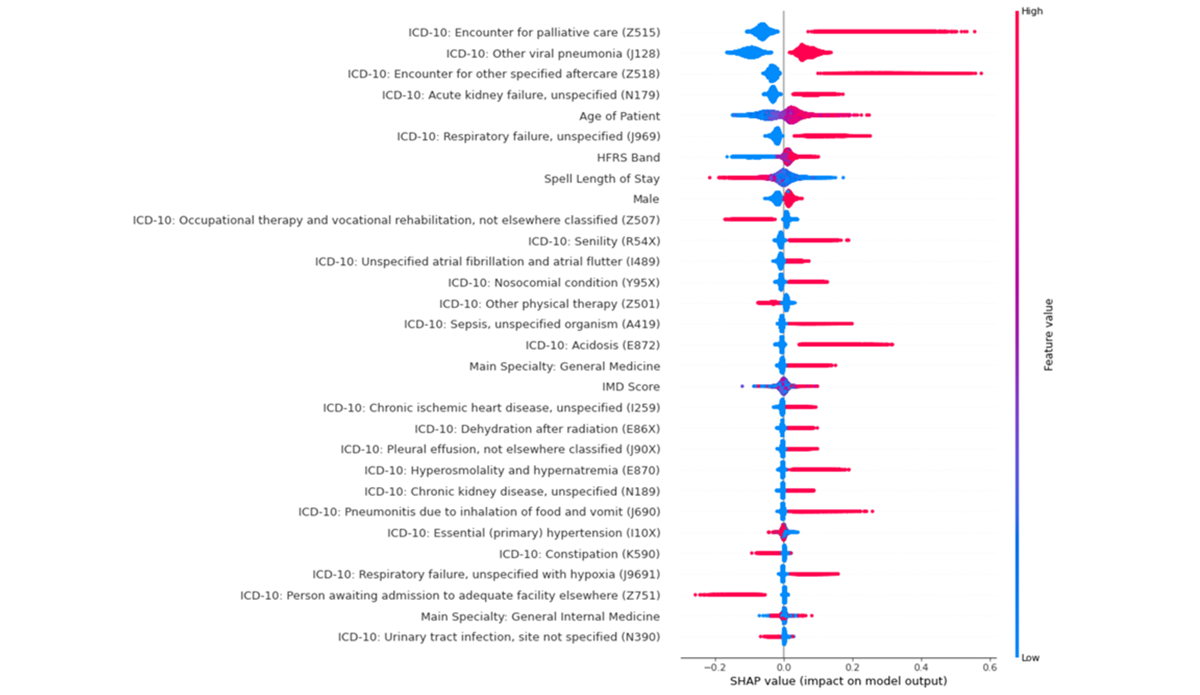
SHAP value dot summary plot for model 2. Each dot in the plot represents a patient. The x-axis indicates whether there is a positive or negative correlation between the value of the feature and its contribution to the model prediction of a patient dying. The color of the dot represents the size of the feature relative to the range of values that feature can take, with red representing large feature values and blue low feature values. The horizontal axis represents the association of the feature value with the outcome. A positive SHAP value means the feature is associated with mortality. A negative SHAP value means the feature contributes to the patient surviving to discharge. The features are ranked by the mean of the absolute value of the SHAP values. HFRS: Hospital Frailty Risk Score; ICD-10: International Statistical Classification of Disease, 10th Edition; IMD: index of multiple deprivation; SHAP: Shapley additive explanation.

Figure 1 shows the SHAP value dot plots for the 30 most important features for model 1. The most important disease severity items that the random forest identified as predictive of mortality were pneumonia, renal failure, and sepsis. The most important frailty items were dementia and delirium, falls and fractures, and pressure ulcers and weight loss. The most important comorbidities were renal disease, heart failure, and primary cancer. Figure 2 shows the probability of in-hospital mortality as calculated by the random forest algorithm as a function of length of stay. In-hospital mortality risk was low for those with length of stay less than 3 days, was relatively stable between 3 and 20 days, and declined with increasing length of stay thereafter. Figure 3 shows the SHAP value dot plots for the 30 most important features for model 2. The HFRS band ranks as one of the most important features.

**Table 3:** Performance of the random forest classifier on the various models. For each model, the random forest classifier accuracy, the area under the receiver operating characteristic curve, and the true positive rate are listed. The true positive rate is the fraction of patients who died who were predicted to have died by the model.

Model	Random forest accuracy, %	Area under the receiver operating characteristic curve, %	True positive rate, %
1	83	90	81
2	82	90	80

Figure S3 in Multimedia Appendix 1 shows the critical care admissions by age band, with the decline in critical care use for older patients reflecting decisions regarding ceilings of care. Figure S4 in Multimedia Appendix 1 shows the time series of the number of hospital admissions and deaths over the course of the study period; higher patient numbers and lower in-hospital mortality rate in the second wave during winter 2020-2021 are apparent. Figures S5 to S7 in Multimedia Appendix 1 are plots of the random forest classifier’s prediction of the probability of mortality as a function of age for patients with and without dementia and delirium, pressure ulcers and weight loss, and falls and fractures. The presence of each domain of frailty was associated with a higher mortality rate for all domains. Figures S8 to S10 in Multimedia Appendix 1 are plots of the random forest classifier’s prediction of the probability of mortality as a function of age for patients with and without cancer, heart failure, and renal disease. Patients with any of these comorbidities had a noticeably higher risk of mortality. Figure S11 in Multimedia Appendix 1 shows the prediction of mortality as a function of age for the 4 HFRS bands and shows the association between greater frailty and in-hospital mortality risk across all age bands.

From the sensitivity analysis, Table S2 in Multimedia Appendix 1 details the AUROC curve for the XGBoost and multivariable logistic regression models. Both models had an AUROC curve of 89%.

## Discussion

Our study is one of very few to use machine learning techniques to explore the role of frailty and comorbidities in COVID-19 outcomes in hospitalized older adults, and by far the largest to date [37]. Measures such as the CFS and HFRS give a global measure of frailty but give little detail on the role of specific aspects of frailty and comorbidity in determining outcomes [38]. As such, their use in guiding decision-making has been questioned [39]. Our study provides a different perspective and explores specific domains of frailty and comorbidities associated with COVID-19 mortality using an administrative data set.

In our study, preexisting dementia, falls and fractures, pressure ulcers and weight loss, renal disease, heart failure, and cancer were all important features in the model.

Dementia/delirium was found to be the most important feature of all the frailty and comorbidity items investigated, with a consistent relationship between dementia/delirium across all ages. Studies from Italy and Brazil have found a higher COVID-19 mortality rate in those with delirium than those without [40,41]. An Italian study of 332 patients found that neurological comorbidities, which included dementia, were associated with a 2-fold increase in mortality, though dementia was not considered in isolation [42].

Various studies have found that patients who have suffered from fractures are at increased risk of dying from COVID-19 [43,44], with one study noting that even though the volume of fracture patients admitted to hospital had decreased during the pandemic, the mortality rate had increased [45]. Respiratory diseases and cardiovascular diseases have been identified as associated with increased COVID-19 mortality risk in other studies [46]. In our study, we identified a substantial increase in the probability of death among patients with falls and fractures compared to those without.

A previous study by members of our team using HES data for all hospitalized adults in England found that all comorbidities in the CCI, except mild liver disease and peptic ulcer, were strong predictors of in-hospital mortality [47]. This is broadly supported by other studies of large administrative databases [48-53].

Age and male sex were important features in all models, which is consistent with previous reports [9,54-56]. The deprivation score was one of the most important features in both our models. Previous studies are inconsistent on the relative importance of deprivation in COVID-19 mortality [57,58]. However, there is a strong relationship between deprivation, ethnicity, age, and other covariates, and it is likely that different modeling approaches address the relationship in different ways.

We found that length of stay had a strong relationship with in-hospital mortality. The risk of death increased between 0 and 3 days before decreasing again after 20 days.

This study has numerous strengths. The use of the HES data set ensures that all hospital activity in England over the first year of the pandemic was captured, minimizing collider bias. We have demonstrated that a random forest classification algorithm is able to predict mortality with reasonable accuracy from an administrative data set. The accuracy of this work can be demonstrated by comparing the true positive rate of model 1 (81%) to the QCOVID risk algorithm, which had a sensitivity of 75.7% for identifying deaths within 97 days in the top 5% of at-risk patients [59]. An external validation of the QCOVID prediction algorithm found the sensitivity in predicting mortality to be 65.94% for men and 71.67% for women in the top 5% of most at-risk patients [60]. Model 1 is clearly comparable to these, despite being trained on an administrative data set lacking clinical details regarding presentation. The risk model for QCOVID used clinical markers for disease severity. It was not our aim to develop a risk prediction algorithm, and we would caution against using our findings to do so, given concerns over data poverty and model accuracy in underrepresented groups (eg, non-White ethnicities). However, provided these concerns can be addressed (eg, through the use of transfer learning in model development [61]), there is clear potential to use large administrative data sets to develop highly accurate models.

There are also limitations to our study, mainly related to the nature of the HES data set. Comorbidities may only be coded if they are deemed relevant to the patient’s condition. As such, the reported prevalence of various domains of frailty and comorbidities is likely to underestimate their true prevalence. For example, it is possible that only the most severe cases of dementia/delirium were recorded in the HES database, which could explain the strong association in our study. Coding of COVID-19 will have been less consistent at the start of the pandemic, particularly with limited testing capacity. For this reason, we included patients diagnosed on clinical grounds, as well as those with a positive test.

We also acknowledge that some secondary diagnoses may have been recorded in the HES database more commonly than others. Issues arise when different trusts’ coding teams code to a different depth of information and when some long-term conditions (eg, diabetes or dementia) are mandatory [62]. We also recognize that in cases of patient transfer to a different trust for treatment, the first admission would have been recorded in our data set as an earlier admission and removed. Thus, the admission period would appear shorter than it actually was. Issues around coding consistency across countries were identified during the GFS development study [20]. This could have impacted the reported relative importance of each frailty/comorbidity feature in the model.

In summary, machine learning has proven useful in understanding the impacts of frailty and comorbidity on mortality. Our findings should help clinicians to identify which COVID-19 patients are most at risk of poor outcomes and help guide treatment strategies during future case surges. Artificial intelligence systems have already found use in guiding treatment strategies for palliative care. [63] A similar approach could be used to triage patients with COVID-19, building on insights from our work.

## Appendix

Multimedia Appendix 1 Supplementary tables and figures.

## Abbreviations

AUROC: area under the receiver operating characteristic
CCI: Charlson Comorbidity Index
CFS: Clinical Frailty Scale
GFS: Global Frailty Scale
HES: Hospital Episodes Statistics
HFRS: Hospital Frailty Risk Score
ICD-10: International Statistical Classification of Disease, 10th Edition
IMD: index of multiple deprivation
LSOA: lower layer super output area
NHS: National Health Service
SHAP: Shapley additive explanation
XGBoost: extreme gradient boosting

## Data Availability

This report does not contain patient identifiable data. Consent from individuals involved in this study was not required. Requests for any underlying data cannot be granted by the authors because the data were acquired from data under license and a data sharing agreement from National Health Service Digital, for which conditions of use and further use apply. Individuals and organizations wishing to access Hospital Episodes Statistics data can make a request directly to National Health Service Digital.
