# Supplementary material for: Frailty, Comorbidity, and Associations With In-Hospital Mortality in Older COVID-19 Patients: Exploratory Study of Administrative Data
Source: Interact J Med Res. 2022 Dec 12;11(2):e41520. doi: 10.2196/41520 (PMC9746678; doi:10.2196/41520)
Supplement: Multimedia Appendix 1 [file ijmr_v11i2e41520_app1.docx]

**Supplementary material Table S1: The hyperparameter ranges explored for the random forest classifier**

| **Hyperparameter** | **Ranges Explored** |
| --- | --- |
| Number of trees | 10-200 |
| Minimum samples per split | 1-20 |
| Minimum Samples per leaf | 1-10 |

**Supplementary material Table S2: Area under the receiver operating characteristic (AUROC) curve for the machine learning algorithms in predicting in-hospital mortality**

| **Model** | **AUROC curve** |
| --- | --- |
| Random forest classifier | 90% |
| XGBoost | 89% |
| Logistic regression | 89% |

**Supplementary Figure S1:** Flowchart of data extraction process. In this figure we display the process by which we generate our dataset starting from the initial dataset of all hospital patients with COVID-19 diagnosis


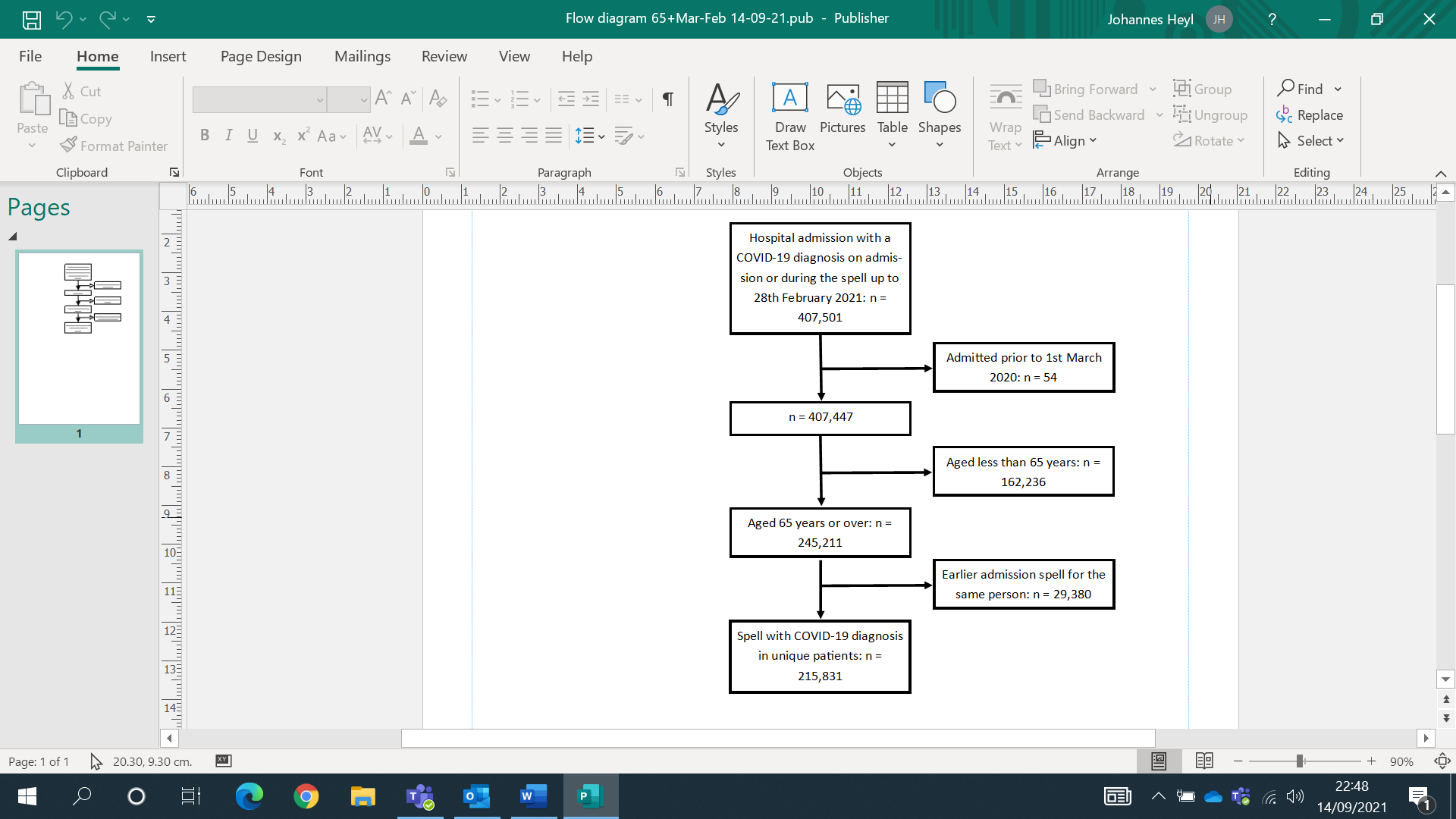


**Supplementary material Figure S2: AUROC curve for Model 1**


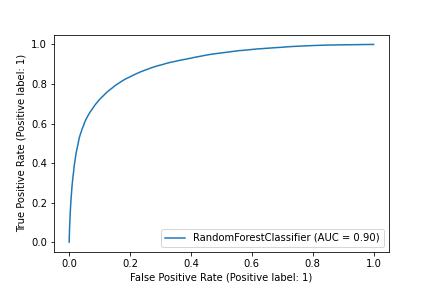


**Supplementary material Figure S3:** Critical care admission rate by age group. We observe that the fraction of each age group in critical care decreases for the older age groups.


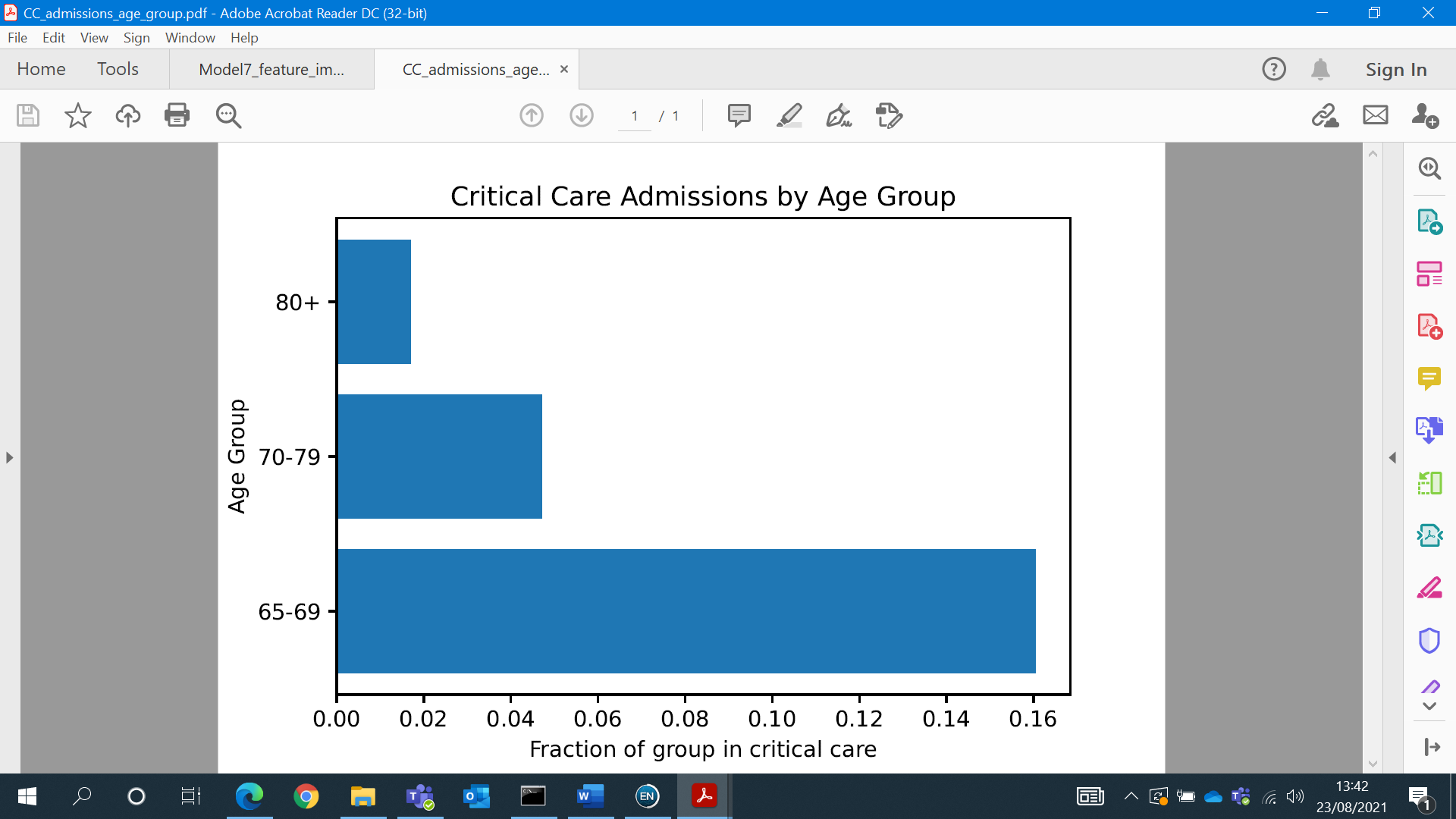


**Supplementary material Figure S4:** Time Series of the Hospital Admissions and Deaths over the 12-month study period. We plot the number of admissions and deaths as a function of time and observe the presence of some peaks in April 2020 and January 2021.

**Supplementary material Figure S5:** A plot of the predicted probability of death as a function of the patient’s age for patients with (blue) and without (orange) the dementia and delirium frailty. The shaded areas represent the 95% confidence interval.

**Supplementary material Figure S6:** A plot of the predicted probability of death as a function of the patient’s age for patients with (blue) and without (orange) the pressure ulcers and weight loss frailty. The shaded areas represent the 95% confidence interval.

**Supplementary material Figure S7:** A plot of the predicted probability of death as a function of the patient’s age for patients with (blue) and without (orange) the falls and fracture frailty. The shaded areas represent the 95% confidence interval.

**Supplementary material Figure S8:** A plot of the predicted probability of death as a function of the patient’s age for patients with (blue) and without (orange) the cancer comorbidity. The shaded areas represent the 95% confidence interval.

**Supplementary material Figure S9:** A plot of the predicted probability of death as a function of the patient’s age for patients with (blue) and without (orange) the heart failure comorbidity. The shaded areas represent the 95% confidence interval.

**Supplementary material Figure S10:** A plot of the predicted probability of death as a function of the patient’s age for patients with (blue) and without (orange) the renal disease comorbidity. The shaded areas represent the 95% confidence interval.

**Supplementary material Figure S11:** A plot of the predicted probability of death as a function of the patient age for patients with the various HFRS bands. The shaded areas represent the 95% confidence interval.
